# Seawater Corrosion Resistance of Zr-Ti Combined Deoxidized Martensitic Stainless Steel

**DOI:** 10.3390/ma18184227

**Published:** 2025-09-09

**Authors:** Qinghai Wu, Shi Cheng, Lei Huang, Xuezhong Huang, Zhihui Wang, Chengyang Hu, Arshad Sundas, Afzal Marina, Barkat Faiqa, Kaiming Wu

**Affiliations:** 1 International Research Institute for Steel Technology, Collaborative Innovation Center for Advanced Steels, Wuhan University of Science and Technology, Wuhan 430081, China; 18277990295@163.com (Q.W.); jxin202212@163.com (Z.W.); arshadsundas@wust.edu.cn (A.S.); m17612771593@163.com (A.M.); mwkd123456@163.com (B.F.); 2 Iron and Steel New Technology Research Institute, Guangxi BG New Materials Co., Ltd., Beihai 536000, China; wust1562023@163.com (L.H.); m15827083619@163.com (X.H.)

**Keywords:** laterite nickel ore, martensitic stainless steel, corrosion-active inclusion, local anti-corrosion resistance property

## Abstract

Martensitic stainless steel is a commonly used stainless steel, and is widely used in daily production and life, but its high content of alloy elements increases its cost. This study aimed to develop low-cost martensitic stainless steel with excellent seawater corrosion resistance by using Zr-Ti combined deoxidation with molten iron from low-priced laterite nickel ore as raw material, taking advantage of the corrosion-resistant elements Cr and Ni, abundant in laterite nickel ore. The characteristics of corrosion-active inclusions in steel, such as their density and electrostatic potential saturation-current density, were observed and studied using scanning electron microscopy and electrochemical testing methods. The intentionally added composite deoxidizing elements (Zr, Ti) form highly stable oxide particles at high temperatures. During the solidification of the molten steel and subsequent solid-state phase transformation, the highly corrosion-active MnS nucleates and disperses on the oxide particles already formed in the liquid phase, while TiN continues to precipitate and coat the MnS particles. This significantly reduces the saturation-current density of locally corrosion-active inclusions, resulting in a marked improvement in seawater corrosion resistance.

## 1. Introduction

With the depletion of conventional iron ore resources, laterite nickel ore, as a surface-layer mineral resource rich in microalloying elements and rare-earth elements, has increasingly attracted attention [1]. Numerous researchers have conducted studies on the smelting and application of laterite nickel ore [2]. Martensitic stainless steel, as a commonly used type of stainless steel, is widely employed in daily production and life. However, there has been no in-depth research conducted by scholars on the possibility of using laterite nickel ore for the smelting and application of martensitic stainless steel resistant to seawater corrosion.

Martensitic stainless steel exhibits excellent corrosion resistance and is primarily used in tool steel, trimming dies, cold-cutting shears, and other ultra-high-strength stainless steels [3,4,5,6,7,8]. In actual use, the presence of Cl^−^ in coastal, marine, and other environments severely damages the passivation film on the surface of stainless steel, which easily induces localized corrosion and defects such as corrosion-active inclusions and micro/nano-sized particles within the steel. This severely disrupts the continuity and uniformity of the stainless steel, significantly impairing the performance of the steel material [9,10,11,12,13,14]. As the most common inclusion in stainless steel, MnS inclusions have an electrode potential lower than that of the passivation film on the surrounding matrix surface, leading them to act as the anodic phase, and dissolve preferentially. The corrosive ions generated by the active dissolution of sulfides further promote the dissolution of the inclusions and the surrounding matrix of the stainless steel [15,16]. Additionally, Williams et al. reported the presence of a 100 nm wide FeS-enriched halo zone around MnS inclusions in stainless steel, suggesting that the dissolution of FeS is the primary cause of pitting corrosion in stainless steel [17]. Wranglen et al. pointed out that inclusions in steel can be classified into active and inactive inclusions. Among these, the sulfur-contaminated zones around sulfide inclusions are more prone to dissolution compared to the matrix, and such inclusions are classified as active inclusions, whereas the matrix around inactive inclusions does not undergo dissolution [18].

By modifying inclusions in steel using different deoxidation methods, it is possible to control the types of inclusions formed in steel, thereby influencing the role of these inclusions in localized corrosion processes. It has been reported that TiN and ZrO_2_ inclusions exhibit better resistance to pitting corrosion than MnS inclusions [19,20,21,22]. Liu Chao et al. utilized CSAFM technology to demonstrate that TiN and ZrO_2_ inclusions are non-conductive, preventing the formation of galvanic corrosion between them and the iron matrix [19]. Wei et al. and Yin et al. utilized first-principles calculations to determine the work functions of different types of inclusions in steel. The results indicated that TiN and ZrO_2_ inclusions have higher work functions than sulfide inclusions and the Fe matrix, suggesting that TiN and ZrO_2_ inclusions are more stable [20,22].

Based on the high content of corrosion-resistant elements Ni and Cr in laterite nickel ore, this paper designs the chemical composition of martensitic stainless steel using laterite nickel-ore smelting iron water as a basis to improve the corrosion resistance of the matrix. Furthermore, in response to localized corrosion induced by large sulfides and oxides in conventional martensitic stainless steel, this study employs Zr-Ti composite deoxidation to replace conventional Al and Si deoxidation, forming finely dispersed composite sulfide-oxide particles. By comparing the localized corrosion resistance of Zr-Ti combined deoxidized steel and conventional martensitic stainless steel in a 3.5% sodium chloride solution (simulating seawater), the corrosion performance of Zr-Ti combined deoxidized steel is evaluated. The electrochemical corrosion behavior induced by corrosion-active inclusions was studied and characterized using optical microscopy, constant potential polarization, and dynamic potential polarization.

## 2. Experimental Materials and Methods

Two martensitic stainless steels with distinct compositions were designed. The ingots were produced via vacuum induction melting using raw materials comprising electrolytic pure iron, high-purity carbon, silicon, manganese, aluminum, chromium, and niobium. These constituents were mixed in specific proportions, melted at elevated temperatures, and thoroughly refined in the vacuum furnace before casting into ingots. Subsequently, the ingots were reheated above 1300 °C and forged into steel bars. Their chemical compositions are detailed in Table 1.

As shown in Table 1, Steel 1 represents conventional martensitic stainless steel composition. Steel 2 was smelted using cast iron derived from laterite nickel-ore-based hot metal, with the Ni content increased. At the same time, Zr, Ti and Nb were added during the LF refining process for composite deoxidation, and a comparative study was carried out.

Microstructural characterization of the experimental steels was conducted at the university’s analytical testing center. Field-emission scanning electron microscope (FE-SEM, AURIGA, Zeiss, Oberkochen, Germany) and Energy-dispersive X-ray spectroscopy (EDS, Genesis 2000, EDAX, Mahwah, NJ, USA) were employed to analyze inclusion morphology and composition using secondary electrons (SE), an accelerating voltage of 10 kV, a working distance of 10 mm, and a beam spot size of 5. Corrosion-active inclusions in high-chloride ion water corrosion solutions can promote the formation of stable pits, induce corrosion pits in the steel substrate, and make the pits more regular and obvious, facilitating counting and statistics. Non-active inclusions do not react, thereby distinguishing corrosion-active inclusions from non-active inclusions. The identification of corrosion-active inclusions in experimental steels followed a custom-developed methodology: a specifically designed corrosive solution contains certain chemical components, such as high-chloride ion water. After polishing the surface of the experimental steels, the corrosive solution was dropped on the sample surface, treated for 5–10 s, and then dried. Samples were subsequently examined under optical microscopy at 100× magnification to quantify the number density of corrosion-active inclusions.

The specimens were cut into samples with a size of 10 × 10 × 10 mm. Five non-exposed surfaces of all electrochemical specimens were encapsulated with epoxy resin, and the area of the exposed surface was 10 × 10 mm. The specimens were progressively ground using silicon carbide abrasive papers up to 1500 grit, followed by mirror polishing with diamond paste (W2.5 grade) and deionized water. Finally, samples were ultrasonically cleaned in anhydrous ethanol, dried, and stored in a desiccator.

Electrochemical testing was performed using a Zahner Zennium workstation (Zahner Elektrik GmbH & Co. KG, Kronach, Germany) employing a standard three-electrode configuration: the experimental steel as working electrode, platinum sheet as counter electrode, and saturated calomel electrode (SCE) as reference electrode (Gao Shrui Lian). The electrochemical tests employed the potentiostatic method to evaluate the resistance to general corrosion under simulated environmental conditions at the maximum saturation-current density. The potentiostatic polarization test was carried out on the experiment steel with reference to the corrosion evaluation standard CTO 00,190,242-003-2017 “Determination of the Saturation Current Density of Anodic Dissolution of Carbon Steel and Low-Alloy Steel in Corrosive Media by Electrochemical Methods” of the Central Research Institute of Ferrous Metallurgy of Russia. The potential for the potentiostatic polarization curve was set at −300 mV, and the test duration was 3600 s. After the test, the maximum value of the saturation-current density, Imax, was recorded. The value ranges of the corrosion stability parameters for each grade are shown in Table 2. The potentiodynamic polarization curves were measured at a scan rate of 0.5 mV/s over a potential range of ±1000 mV, relative to the open-circuit potential (OCP). The test solution used was 3.5 wt.% NaCl solution. In addition, the electrochemical-impedance spectroscopy test frequency range was 100 Khz~10 mHz, the amplitude was 10 mV, and the results were analyzed using Zsimpwin software (Version 3.6).

Micro-area electrochemical testing was conducted using a scanning vibrating electrode technology (SVET) system (Versascan, Ametek, Berwyn, PA, USA) to obtain localized corrosion-current density profiles of samples immersed in NaCl solution for varying durations. An electrometer equipped with the Versascan was used to measure the local potential difference across the sample surface. The potential difference was converted to current density using the formula I = −ΔE/R (solution resistance R = d/k = 3.563 × 10^−5^ Ω, where d is the probe vibration amplitude perpendicular to the sample, which is 30 μm, and k is the solution conductivity, which is 8.42 mS/cm). A Pt–Ir probe with a diameter of approximately 5 μm was used, and the vibration frequency was 80 Hz.

## 3. Result

### 3.1. The Formation of Complex Inclusions

**Figure 1:**
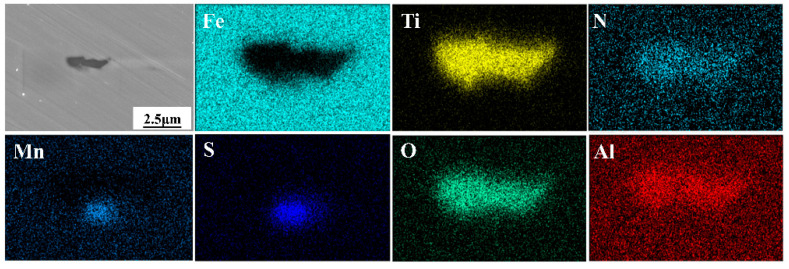
Characterization of inclusions in conventional martensitic stainless steels.

**Figure 2:**
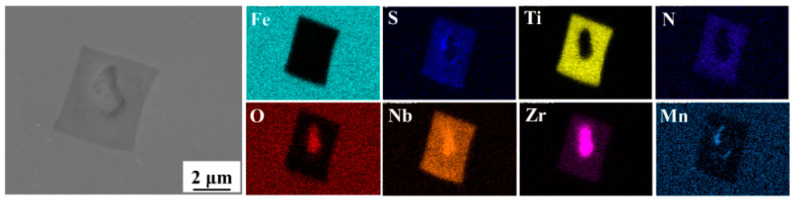
Characterization of inclusions in Zr-Ti combined deoxidized martensitic stainless steel.

The morphology of inclusions in conventional martensitic stainless steel and complex deoxidized martensitic stainless steel was observed using SEM, with EDS analysis used to determine the composition of the inclusions. Figure 1 shows that the inclusions in conventional martensitic stainless steel exhibit an elongated shape. EDS spectra revealed that the distributions of Ti and N are consistent, those of Al and O are consistent, and those of Mn and S are consistent. Therefore, it is inferred that the inclusions in conventional martensitic stainless steel are composite inclusions composed of Al_2_O_3_ + MnS + TiN, with particle sizes approximately 3–5 μm. As shown in Figure 2, the inclusions in Zr-Ti combined deoxidized martensitic stainless steel exhibit a complex morphology, with ellipsoidal inclusion cores enclosed by square-shaped inclusion shells. The corresponding EDS spectra confirm that the distributions of Zr, Nb, and O are consistent, the distributions of Ti, Nb, N, and S are consistent, and the distributions of Mn and S are consistent. Therefore, it is inferred that the inclusions in the Zr-Ti combined deoxidized martensitic stainless steel are a multi-component composite inclusion, consisting of (Ti,Nb)-N-S + (Zr,Nb)-O + MnS, with the inclusion size being approximately 4 μm.

### 3.2. Corrosion-Active Inclusions

**Figure 3:**
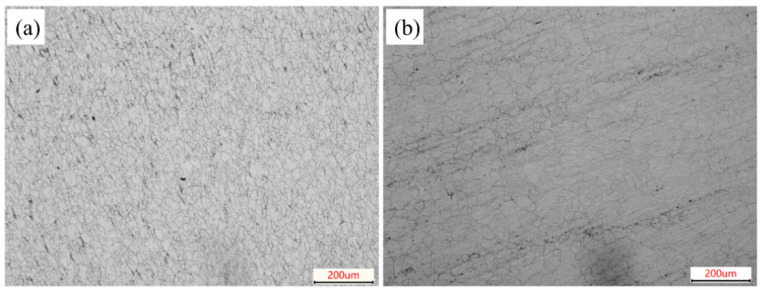
Optical micrographs of corrosion-active inclusions. (**a**) Conventional martensitic stainless steels; (**b**) Zr-Ti combined deoxidized martensitic stainless steels.

Corrosion-active inclusions tests were carried out on conventional and composite-deoxidized martensitic stainless steels [23]. The microstructural micrographs after corrosion are shown in Figure 3. Additionally, a statistical analysis was performed on the density of corrosion-active inclusions in both conventional and composite-deoxidized martensitic stainless steels. On the surface of conventional martensitic stainless steel, black dots (pitting corrosion craters) were observed, resulting from the dissolution of corrosion-active inclusions [24]. The areal density of corrosion-active inclusions on both sample surfaces was subsequently analyzed via statistical methods. The density of corrosion-active inclusions in conventional martensitic stainless steel is 0.515 (±0.056) particles/mm^2^, while that in composite-deoxidized martensitic stainless steel is 0.047 (±0.015) particles/mm^2^. Statistical analysis reveals that composite-deoxidized martensitic stainless steel smelted from laterite nickel ore and treated with Zr and Ti exhibits a significantly lower density of corrosion-active inclusions compared to conventional martensitic stainless steel [25]. Literature reports indicate that MnS inclusions—either individually or in their surrounding microregions—are highly prone to dissolution in NaCl solutions, thereby inducing pitting corrosion and demonstrating elevated corrosion activity [14]. Following Zr-Ti composite deoxidation, the originally highly corrosive MnS inclusions in the steel matrix are transformed into Zr/Ti-bearing composite inclusions (e.g., ZrO_2_, TiN). These modified inclusions exhibit superior chemical stability and corrosion resistance in NaCl environments, consequently reducing the overall density of corrosion-active inclusions in composite-deoxidized martensitic stainless steel [26].

### 3.3. Potentiostatic Polarization

**Figure 4:**
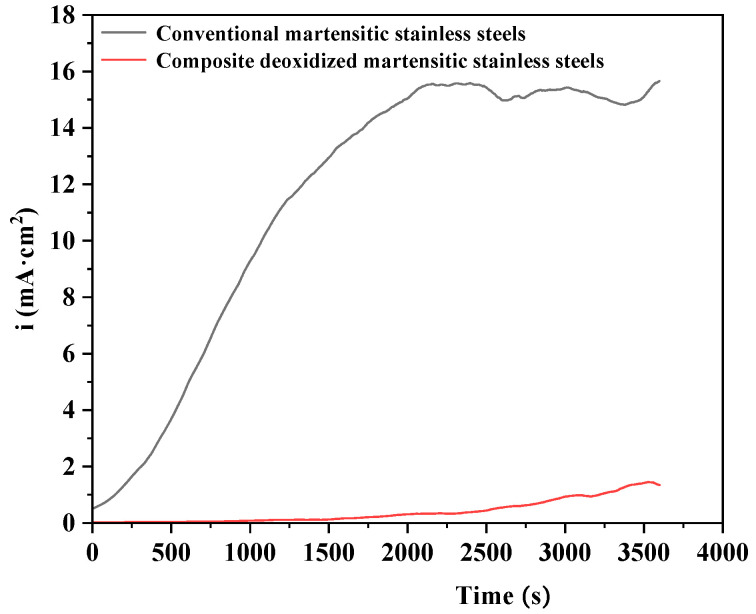
Potentiostatic polarization curve of two martensitic stainless steels.

Electrochemical potentiostatic polarization tests were performed on both conventional and composite-deoxidized martensitic stainless steel specimens. The resulting saturation-current density profiles are depicted in Figure 4. For equivalent exposure durations, lower saturation-current density values are indicative of enhanced corrosion resistance in the composite-deoxidized martensitic stainless steel. The saturation-current density of conventional martensitic stainless steel is 15.26 mA·cm^−2^, while that of composite-deoxidized martensitic stainless steel is 1.44 mA·cm^−2^ —a reduction of 13.82 mA·cm^−2^ for the composite-deoxidized variant. Thus, the development of composite-deoxidized martensitic stainless steel using laterite nickel ore, combined with multi-element composite deoxidation (e.g., Zr and Ti), effectively alters the types and morphologies of inclusions, reduces the density of corrosion-active inclusions, significantly lowers the saturation-current density, and enhances the corrosion resistance of the steel [27].

### 3.4. Electrochemical Impedance Spectroscopy (EIS) Testing

**Figure 5:**
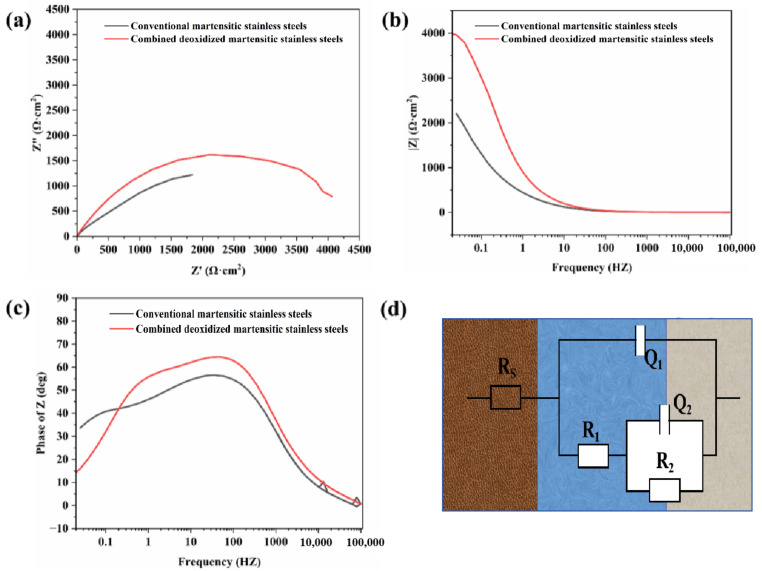
Electrochemical impedance diagrams of two types of martensitic stainless steel. (**a**) Nyquist diagram, (**b**,**c**) Porter diagram, (**d**) Equivalent circuit diagram.

To further investigate the characteristics of the passivation films formed on the surfaces of the two martensitic stainless steels, the EIS tests were conducted at open-circuit potential in a 3.5 mol/L NaCl solution (at room temperature). Figure 5 shows the Nyquist plots and Bode plots of the conventional martensitic stainless steel and the composite deoxidized martensitic stainless steel in a 3.5 wt.% NaCl solution. From the Nyquist plots, it can be observed that the curves of both steels exhibit a typical semicircular arc, with the composite deoxidized martensitic stainless steel having the largest diameter and the traditional martensitic stainless steel having the smallest diameter. The similar capacitive arc features in the Nyquist plots indicate that they have similar passivation mechanisms. The diameter of the capacitive arc reflects the electronic transfer resistance on the sample surface; generally, a larger diameter indicates higher membrane resistance and stronger corrosion resistance. In the Bode plots, a larger impedance modulus |Z| in the low-frequency region (0.01–0.1 Hz) indicates better corrosion resistance [22]. As shown in Figure 5b, the |Z| value in the low-frequency region of the Bode plot for composite deoxidized martensitic stainless steel slightly increases, indicating enhanced corrosion resistance, consistent with the results from the Nyquist plot. Additionally, the phase angle (-Phase) varies between 40 and 80°, indicating that the passivation film is primarily capacitive. The phase angle shows a slow increase in the mid-to-high frequency range (1–102 Hz), indicating that the passivation film is relatively stable under these conditions. After the specimen undergoes stable pitting corrosion, the time constants exhibit a noticeable separation. Although the Bode curves of the two stainless steels are very similar, it can still be observed that, at the same frequency, the composite deoxidized martensitic stainless steel has a slightly higher impedance (|Z|) and a slightly larger phase angle (-Phase). 

Equivalent circuits were used to analyze the EIS data of two martensitic stainless steels in a 3.5 mol/L NaCl solution. Considering that a passivation film forms after open-circuit potential testing of the samples, combined with electrochemical corrosion mechanisms, the porous structure of the passivation film, and fitting errors, the equivalent circuit shown in Figure 5d was ultimately adopted. In this circuit, Rs represents the solution resistance, and Q1 and R1 represent the capacitance and resistance of the double layer, respectively, while Q2 and R2 are related to the corresponding properties of the passivation film [13]. According to the fitting results (Table 3), R2 is significantly larger than R1, indicating that R2 is the primary resistance against corrosion and plays a crucial role in the charge transfer process of the passivation film. The increased value of R2 in the new martensitic stainless steel suggests that a more corrosion-resistant passivation film has formed on the surface of the composite deoxidized martensitic stainless steel. The phase angle index n reflects the degree of deviation between the actual capacitance and the ideal capacitance. The larger the value of n, the smaller the deviation from the ideal capacitance. Generally, the n value of the double layer capacitance of corroded electrodes is between 0.5 and 1. The value is larger in deoxidized martensitic stainless steel, indicating that its value is more reliable.

### 3.5. Potentiodynamic Polarization

**Figure 6:**
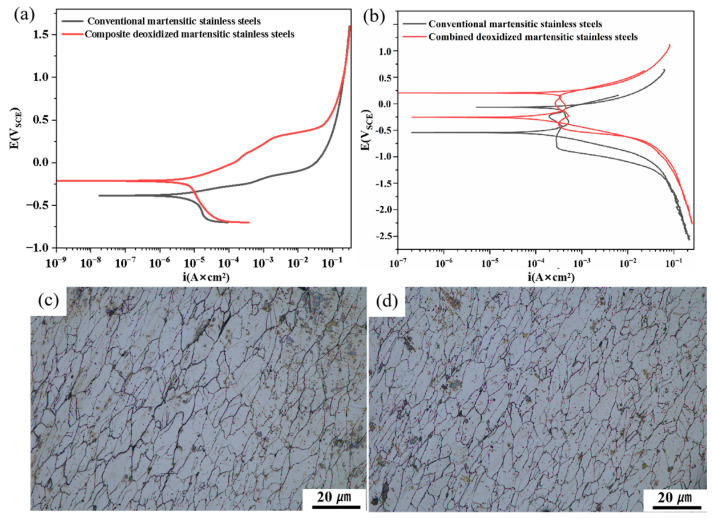
Dynamic potential polarization curves (**a**), cyclic polarization curves (**b**), and morphology images (**c**,**d**) after cyclic polarization testing of two martensitic stainless steels.

Figure 6a presents the potentiodynamic polarization curves of conventional and composite-deoxidized martensitic stainless steels in a 3.5 wt.% NaCl solution. It is evident from the curves that neither steel exhibits a passivation region, indicating active dissolution behavior [28]. The Tafel extrapolation method was employed to fit the potentiodynamic polarization curves, yielding the corrosion potential (E_corr_) and corrosion-current density (I_corr_) for both samples [29]. These parameters were used to evaluate the corrosion rate trends and reaction mechanisms. The corresponding E_corr_ and I_corr_ values are listed in Table 4. The corrosion potential (E_corr_) values of the conventional and composite-deoxidized martensitic stainless steels are −0.385 V and −0.212 V, respectively, while their corrosion-current density (I_corr_) values are 4.75 × 10^−6^ A·cm^−2^ and 3.93 × 10^−6^ A·cm^−2^, respectively. The results show that the corrosion potential (E_corr_) of the composite-deoxidized martensitic stainless steel is higher than that of the conventional martensitic stainless steel, while its corrosion-current density (I_corr_) is lower. A more positive E_corr_ indicates better thermodynamic stability of the metal and a lower tendency for corrosion. A smaller I_corr_ value signifies a slower corrosion rate. With a smaller I_corr_ and a more positive E_corr_, the composite-deoxidized martensitic stainless steel exhibits superior corrosion resistance compared to the conventional counterpart. Faraday’s law can be used to calculate corrosion rates [30]. The penetration rate (CR) was calculated for two different stainless steels (Table 4), demonstrating that Zr-Ti combined deoxidized martensitic stainless steels exhibit lower corrosion rates. The overall shapes of the dynamic potential polarization curves for the two samples in the cathode (reduction reaction) and anode (oxidation reaction) branches are similar. Due to the evolution of water and dissolved oxygen, the dynamic potential polarization curve exhibits a horizontal curve at the lowest potential (−0.75 V) in the cathode branch. Within this potential range, part of the potential is utilized for subsequent reactions. Additionally, the cathode branch curve exhibits a slow decline followed by a vertically inclined section, indicating that the reduction reaction is mixed-controlled (diffusion and activation). The anode branch curve rises rapidly, indicating that the oxidation reaction is activation-controlled and that the material has high chemical activity.

Figure 6b shows the cyclic potentiodynamic polarization curves of conventional and composite deoxidized martensitic stainless steel in a 3.5 wt.% NaCl solution. The composite deoxidized martensitic stainless steel exhibits a higher pitting breakdown potential (Eb), indicating that it requires a higher driving force to initiate pitting corrosion, and its repassivation potential (Eprot) is also significantly more positive. The reverse scan curve intersects with the forward scan curve at a more positive potential, forming a smaller hysteresis loop, indicating that once pits form, they can rapidly re-passivate as the potential decreases, resulting in a lower risk of pitting-corrosion propagation. In contrast, conventional martensitic stainless steel performs poorly, with a significantly more negative Eb value, indicating that pitting corrosion occurs at lower potentials. More critically, its reverse scan curve intersects with the forward scan curve to form a large and wide hysteresis loop, with a very negative Eprot value. This clearly indicates that the material not only tends to undergo pitting corrosion but, once pitting occurs, it is difficult to stop, with the pits continuing to grow and expand until severe localized corrosion occurs. Additionally, the morphology images after cyclic dynamic potential polarization testing (Figure 6c,d) show that conventional martensitic stainless steel exhibits significant pitting corrosion after electrochemical testing. In contrast, composite deoxidized martensitic stainless steel exhibits less pitting corrosion, with the substrate largely preserved in its original state, demonstrating better corrosion resistance.

### 3.6. Localized Corrosion Experiment

**Figure 7:**
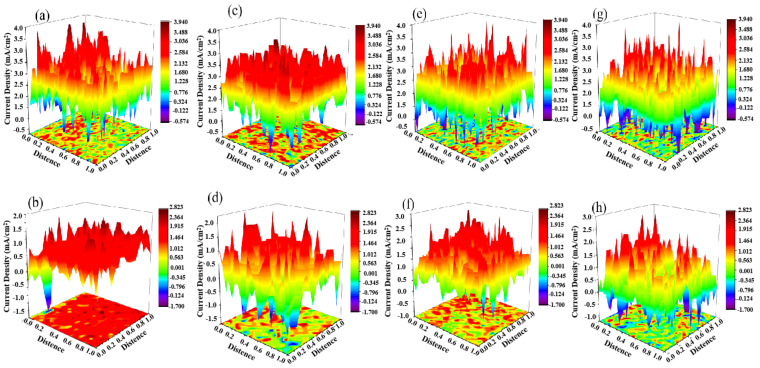
Distribution of SVET on the surfaces of two types of martensitic stainless steel: (**a**,**c**,**e**,**g**): Corrosion of conventional martensitic stainless steel after 30, 60, 90, and 120 min; (**b**,**d**,**f**,**h**): Corrosion of combined deoxidized martensitic stainless steels after 30, 60, 90, and 120 min.

The in situ corrosion behavior of two martensitic stainless steels undergoing spontaneous corrosion in a 3.5 wt.% NaCl solution was observed using SVET. The current distribution diagrams at immersion times of 30, 60, 90, and 120 min are shown in Figure 7. After immersion for a certain period of time, both steels exhibited high anodic current density peaks, indicating that the electron transfer and ion migration processes on the corrosion-affected surface generate strong anodic current signals. This is primarily due to the spontaneous dissolution of the inclusion regions in the steel within the NaCl solution [31]. Long-term immersion SVET results showed that anodic current density peaks still existed at extended immersion times, indicating that both stainless steels exhibited localized corrosion in NaCl solution over extended periods. Under high localized electrochemical activity, the corrosion did not propagate, demonstrating that the matrix structure possesses a well-developed corrosion-resistant passivation film [32]. However, conventional martensitic stainless steels exhibit more anodic current density peaks, indicating that conventional martensitic stainless steels have higher corrosion-active inclusions.

**Figure 8:**
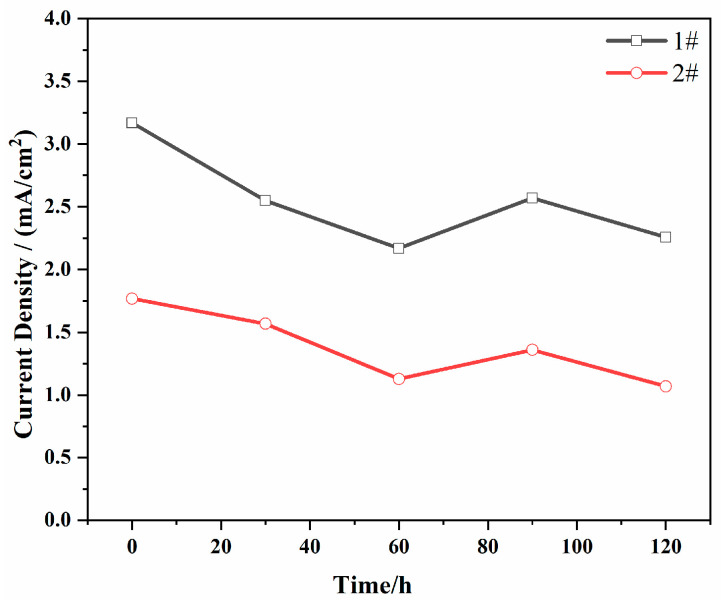
Changes in corrosion-current density over time for two types of martensitic stainless steel.

Further processing of the data reveals the change in corrosion-current density of the test steel samples over time, as shown in Figure 8. After 120 min of corrosion testing, the corrosion-current density of the steel tends to stabilize, indicating that at different time points, the corrosion-current density of the combined deoxidized martensitic stainless steels is lower than that of conventional martensitic stainless steel. The corrosion-current density of conventional martensitic stainless steel stabilizes at 2.37 mA/cm^2^, while that of the combined deoxidized martensitic stainless steels stabilizes at 1.04 mA/cm^2^. A higher corrosion-current density indicates a faster corrosion rate and poorer corrosion resistance. In comparison, the corrosion resistance of the combined deoxidized martensitic stainless steels is superior to that of conventional martensitic stainless steel.

**Figure 9:**
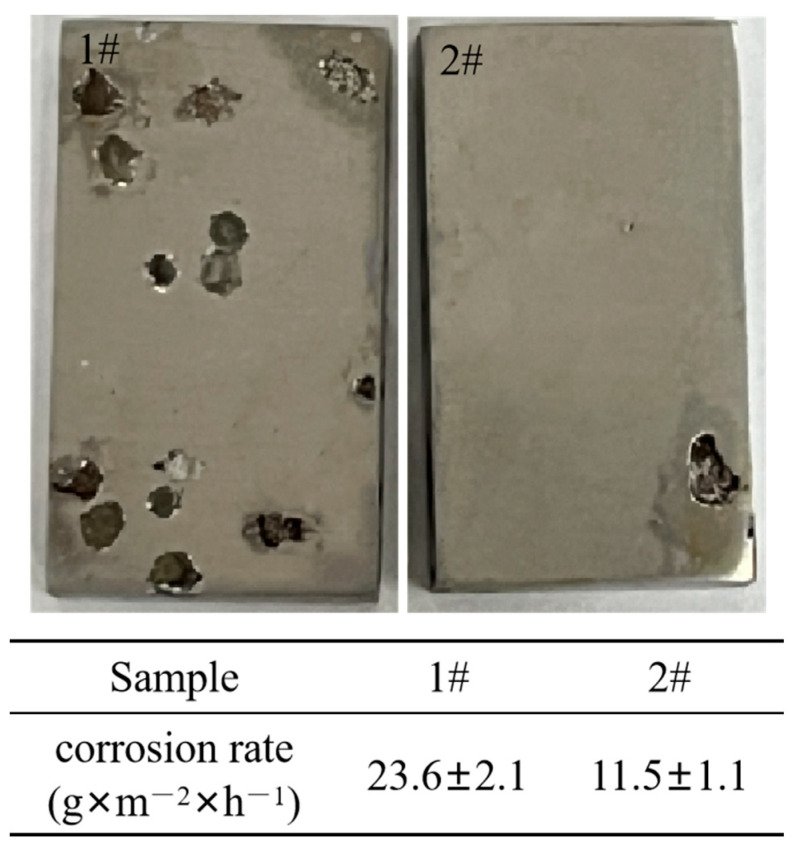
Macrographs of the two types of stainless steel after immersion in a 6% FeCl_3_ solution and corrosion rates: 1# conventional martensitic stainless steel, 2# composite deoxidized martensitic stainless steel.

To visually characterize the corrosion rates of the two steels, macroscopic surface-morphology images and corrosion rates were obtained after immersing the samples in a 6% FeCl_3_ solution for 24 h (Figure 9). By weighing the samples before and after corrosion and recording the surface area of the specimens, the average corrosion rates for the two groups of experimental steels were calculated. As shown in Figure 9, the traditional martensitic stainless steel exhibits a higher number of pitting corrosion pits with larger dimensions, indicating poorer corrosion resistance. In contrast, the composite deoxidized martensitic stainless steel specimens exhibit lighter corrosion and lower corrosion rates.

## 4. Discussion

### 4.1. The Effect of Composite Deoxidation on Inclusion Formation in Steel

According to fundamental principles of metallurgical thermodynamics, Zr and Ti are both strong oxide-forming elements. Composite deoxidation with Zr and Ti facilitates the removal of free oxygen content in molten steel. The densities of ZrO_2_ are 5.68 g/cm^3^, respectively, which are higher than that of Al_2_O_3_ (3.97 g/cm^3^). Notably, ZrO_2_ has a density (5.68 g/cm^3^) closer to that of molten steel (7.15 g/cm^3^) [31]. Thus, once stable oxides form at high temperatures, ZrO_2_ can uniformly float in the molten steel. In contrast, Al_2_O_3_ tends to collide and agglomerate at the surface of the molten steel to become part of the slag [33]. The large clustered Al_2_O_3_ inclusions may remain in the steel if they fail to float up, where the electrical conductivity of oxides is critical to their movement in molten steel. Previous studies have shown that the driving force for Al_2_O_3_ movement in molten steel is greater than that for ZrO_2_ [34]. During refining ZrO_2_ particles tend to repel each other and hardly agglomerate, while Al_2_O_3_ easily collides to form large particles that float to the molten steel surface and are absorbed by the molten steel-surface covering agent [35]. Therefore, compared with conventional Al-Si deoxidation, Zr-Ti deoxidation can form fine and dispersed composite oxides, as demonstrated by the experimental results in Figure 1 and Figure 2.

ZrO_2_ is a cation-vacancy oxide, and partial metal Mn atoms can diffuse into the cation vacancies of ZrO_2_. As is well known, Mn and S in steel have a strong affinity. Therefore, the formation of ZrO_2_ particles facilitates the precipitation of MnS on their surfaces. Additionally, literature reports indicate that MnS and ZrO_2_ have extremely similar lattice constants [36,37] (see Table 5). It can be seen that ZrO_2_ and MnS exhibit good lattice matching, which significantly reduces the interfacial energy between them and promotes the precipitation of MnS on ZrO_2_ formed at high temperatures. The experimental results in Figure 1 and Figure 2 also confirm this.

### 4.2. The Effect of Composite Microalloying on Localized Corrosion of Martensitic Stainless Steel

To compare the corrosion resistance differences between traditional and combined deoxidized martensitic stainless steels, EIS and dynamic potential polarization curve tests were conducted. The results show that the corrosion-current density in the combined deoxidized martensitic stainless steels is significantly lower than that in traditional martensitic stainless steel, indicating that the addition of elements such as Ni, Zr, and Ti can significantly enhance the corrosion resistance of stainless steel. Elements such as Zr and Ti have strong metallic properties, and preferentially combine with S and O to form oxygen sulfides that precipitate from the molten steel, thereby purifying the molten steel. They can also alter the morphology of oxygen and sulfur inclusions formed in the steel, reducing the number of harmful inclusions [38]. Additionally, Ni is an effective element for enhancing the corrosion resistance of steel, typically existing in the passivation film in the form of +2 valence NiFe_2_O_4_, which increases passivation stability and improves the steel’s corrosion resistance [39].

For the Al_2_O_3_ + MnS + TiN composite inclusions in conventional martensitic stainless steel, the MnS inclusions exhibit conductivity and can form galvanic corrosion with the surrounding Fe matrix. Additionally, due to its lower volt potential compared to the surrounding Fe matrix, the MnS inclusion acts as an anode and undergoes dissolution, preferentially in galvanic corrosion [40]. As the dissolution reaction of MnS inclusions proceeds, a locally acidic environment rich in sulfur forms around them, further accelerating the dissolution of the inclusions and the surrounding Fe matrix, ultimately leading to the formation of corrosion pits on the surface. Therefore, MnS inclusions exhibit high corrosion activity, increasing the corrosion activity inclusion density and corrosion rate of conventional martensitic stainless steel. By modifying the inclusions through the addition of Zr, Ti, and Nb elements, the primary types of inclusions in composite deoxidized martensitic stainless steel are (Ti, Nb)-N-S + (Zr, Nb)-O + MnS, with MnS inclusions located at the core of the inclusions and surrounded by (Ti, Nb)-N-S inclusions. The conductive MnS inclusions are separated from the Fe matrix by the non-conductive (Ti, Nb)-N-S inclusions, preventing the formation of galvanic corrosion between the MnS inclusions and the Fe matrix [21]. According to the research results of Liu et al., ZrO_2_ inclusions are non-conductive and cannot form galvanic corrosion with the Fe matrix. In another study, it was also found that TiN inclusions act as a cathodic phase and are protected in the formed galvanic corrosion. The E-pH phase diagram of the Fe-Zr-Ti-H_2_O system also shows that ZrO_2_ inclusions and TiN inclusions are relatively stable in the studied solution system [21]. Therefore, after composite deoxidation with elements such as Zr and Ti, the corrosion activity of inclusions in martensitic stainless steel is significantly reduced, resulting in a marked decrease in the density of corrosion-active inclusions and corrosion rate. In localized corrosion, they exhibit lower anodic current density peaks (Figure 7) and demonstrate superior corrosion resistance.

As shown in the constant potential polarization curve (Figure 4), the current density values of the conventional martensitic stainless steel exhibit a gradual decrease trend between 0 and 1500 s, indicating that a passivation film forms on the sample surface during this time period, and the nucleation and growth rates of the passivation film exceed the dissolution rate. After 1500 s, the current density suddenly increases, suggesting that the passivation film formed over time is disrupted and undergoes dissolution. In contrast, the current density of traditional martensitic stainless steel increases from the beginning, indicating the instability of its passivation film. Additionally, the EIS diagram shows that the composite deoxidized martensitic stainless steel has high impedance (|Z|) values and phase angles (-Phase). This indicates that the passivation film of the composite deoxidized martensitic stainless steel is more stable under these conditions. The cyclic polarization data in Figure 6 also show that, during the early stages of electrochemical corrosion, both types of martensitic stainless steel exhibit primarily pitting corrosion. As the experiment progresses, passivation occurs on the experimental surface. Surface morphology images reveal that traditional martensitic stainless steel exhibits a higher degree of pitting corrosion, which later spreads to more significant general corrosion. This is primarily due to the higher density of corrosion-active inclusions in traditional martensitic stainless steel, resulting in more pitting-corrosion pits during the early stages of electrochemical corrosion. Additionally, research has shown that an increase in nickel content helps form a more stable passivation film [41]. The introduction of high nickel content in composite deoxidized martensitic stainless steel results in a more stable surface passivation film, with a lower diffusion rate of general corrosion in the later stages (Figure 9). Therefore, the high corrosion resistance of composite deoxidized martensitic stainless steel is the combined result of improved corrosion-active inclusions and enhanced passivation film stability, due to increased nickel content.

## 5. Conclusions

(1) Compared with conventional martensitic stainless steel, composite-deoxidized martensitic stainless steel exhibits significantly reduced saturated-current density at constant electrode potential and superior seawater corrosion resistance. This is primarily attributed to the deliberate modification of corrosion-active inclusions by added composite deoxidizing elements, which induces notable changes in their microstructure and morphology. Specifically, highly corrosion-active MnS is encapsulated by TiN, leading to a substantial reduction in the density of corrosion-active inclusions and a marked improvement in resistance to localized seawater corrosion.

(2) The composite-deoxidized martensitic stainless steel exhibits a lower corrosion-current density (I_corr_) and a more positive corrosion potential (E_corr_) in potentiodynamic polarization tests compared to conventional martensitic stainless steel, indicating its superior resistance to seawater corrosion. This enhancement is attributed to the incorporation of Ni and Cr from laterite nickel ore, which elevate the matrix’s intrinsic corrosion resistance and thereby contribute to the observed improvement in seawater corrosion performance.

## Figures and Tables

**Table 1:** Chemical composition of the experiment steel/wt.%.

	C	Si	Mn	P	S	Cr	Ni	Zr + Ti	Nb	Fe
1	0.30	0.52	0.49	0.0066	0.0045	13.43	0.48	-	-	Bal.
2	0.29	0.56	0.48	0.0080	0.0050	13.78	1.40	0.005–0.060	0.009–0.030	Bal.

**Table 2:** The parameter values of corrosion stability levels 1, 2, and 3.

Anti-Corrosion Grade	I_max_, mA/cm^2^
1 High corrosion stability	<3
2 Qualified corrosion stability	3–6
3 Unstable corrosion stability	>6

**Table 3:** EIS electrochemical fitting parameters for two types of martensitic stainless steel.

Sample	R_s_ (Ω·cm^2^)	Q_1_ (10^4^Ω^−1^ s^n^cm^−2^)	n_1_	R_1_ (Ω·cm^2^)	Q_2_ (10^4^Ω^−1^ s^n^cm^−2^)	n_2_	R_2_ (Ω·cm^2^)	Chsq
Conventional martensitic stainless steel	4.36	2.83	0.83	5.32	2.95	0.85	3.12 × 10^3^	4.31 × 10^−3^
Composite deoxidized martensitic stainless steel	4.78	1.05	0.91	7.12	1.43	0.93	5.81 × 10^3^	8.35 × 10^−4^

**Table 4:** Fitting results of potentiodynamic polarization of two martensitic stainless steels.

	i(A·cm^−2^)	E(V, vs. SCE)	CR(mm/yr)
**conventional martensitic stainless steels**	4.75 × 10^−6^	−0.385	0.0326
**Zr-Ti combined deoxidized martensitic stainless steels**	3.93 × 10^−6^	−0.212	0.0269

**Table 5:** Lattice parameters of MnS and ZrO_2_ [37].

Category	Crystal Structure	Planes (hkl)	Plane Distance	β	int	h	k	l	a	b	c
ZrO_2_	Monoclinic	002	2.621	99.23	20	0	0	2	5.145	5.207	5.311
		022	1.847		14	0	2	2			
		113	1.509		4	1	1	3			
MnS	Fcc	111	2.612	90	100	1	1	1	5.224	5.224	5.224
		220	1.847		50	2	2	0			
		222	1.509		20	2	2	2			

## Data Availability

The original contributions presented in this study are included in the article. Further inquiries can be directed to the corresponding authors.
